# Comparative Evaluation of Oven, Microwave, and Air-Fry Roasting on Physical Attributes, Bioactive Components, and Volatile Compounds of Rosemary- and Thyme-Coated Mung Bean *(* *Vigna radiata* *L.)*

**DOI:** 10.1021/acsomega.6c00029

**Published:** 2026-05-20

**Authors:** Ahsen Rayman Ergun, Kadriye Altay, Yunus Çetintaş, Yeliz Tekgül Barut

**Affiliations:** † 37509 Ege University, Faculty of Engineering, Food Engineering Department, İzmir 35040, Turkiye; ‡ Ministry of Agriculture and Forestry, Olive Research Institute, Izmir 35100, Turkiye; § Food Analysis Application and Research Center, Research Laboratories Center, 52986 Mugla Sitki Kocman University, Muğla 48000, Türkiye; ∥ Food Processing Department, Kosk Vocational School, Adnan Menderes University, Aydın 09100, Türkiye

## Abstract

The increasing demand for plant-based proteins has intensified interest in alternative legume sources with high nutritional and functional value. Mung bean is a promising protein source; however, limited information is available about the effects of edible coatings and different roasting methods on its quality characteristics. This study investigated the impact of rosemary- and thyme-based coatings combined with oven, microwave, and air-fry roasting on the physicochemical, bioactive, and volatile properties of a high-protein mung bean snack. Rosemary and thyme were chosen as edible coatings because of their well-documented antioxidant and aroma-enhancing properties, making them suitable for improving the functional and sensory quality of legume-based snacks. Coated mung beans were roasted using oven (200 °C, 20 min), microwave (600 W, 8 min), and air-fry (200 °C, 13 min) methods, with the final moisture content maintained below 6%. Moisture, ash, protein, total phenolic and flavonoid contents, antioxidant activity, color parameters, and volatile compounds were evaluated. Air-fried samples exhibited higher phenolic, flavonoid, and antioxidant contents than those of oven- and microwave-roasted samples. Especially, in the rosemary-microwave sample group, the phenolic content was found to be 1447.0 ± 20.02 mg GAE/100 g DM. The highest protein content (25.96%) was observed in the air-fried rosemary-coated samples. Lightness decreased after roasting, while a* and b* values were largely preserved. Coating type and roasting method significantly influenced the volatile profiles, with rosemary- and thyme-coated samples showing higher terpene contents, predominantly limonene. These findings indicate that edible coatings combined with alternative roasting technologies can enhance the nutritional and sensory quality of mung bean-based snacks.

## Graphical abstract

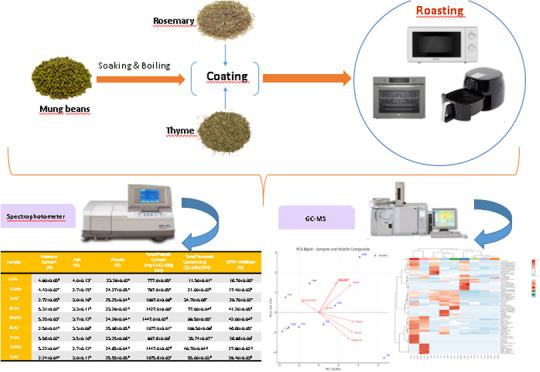

## Practical Implications

Mung bean could be widely utilized in the snack food industry in various forms, including extruded mung bean chips, baked legume-based snacks, high-protein crackers and grissini, gluten-free puffed snacks, as well as vegan and clean-label products, due to its favorable nutritional profile and functional properties. Determining the effects of the roasting method on the bioactive compounds is important for the industry. So this paper enhances further studies and novel products.

## Introduction

Mung bean (*Vigna radiata* L.) is an excellent plant-based protein source, containing approximately 27% protein, with an essential amino acid profile comparable to soybean, kidney bean, and the FAO/WHO reference protein.^1^ In addition to its high protein content, mung beans provide 59–65% carbohydrates, 1–1.5% fat, 3.5–4.5% dietary fiber, and 4.5–5.5% ash on a dry weight basis, supplying about 3400 kcal kg^–1^ of energy.^2^ Consumption of mung beans has been associated with beneficial health effects, including a low postprandial glycemic response in humans and improvements in glucose and lipid metabolism in animal studies.^3^ Despite these nutritional and health-promoting attributes, the use of mung beans in value-added snack products remains limited. This limitation is largely associated with changes in sensory quality, nutritional composition, and stability of bioactive compounds during thermal processing. Although cooking and roasting can improve digestibility and palatability, inappropriate processing conditions may negatively affect functional components, emphasizing the need for alternative processing strategies that preserve chemical quality while maintaining consumer acceptability.

The incorporation of herbs and spices as natural antioxidants to inhibit oxidative reactions and enhance food quality has gained increasing attention in recent years. Among these, rosemary and thyme are widely studied due to their strong antioxidant capacity, which is mainly attributed to their high content of phenolic compounds, including diterpenes, flavonoids, and phenolic acids such as rosmarinic and carnosic acids.^4^ Beyond their antioxidant activity, these herbs exhibit various bioactive properties, including antibacterial, anti-inflammatory, antifungal, antiseptic, anticancer, and antimutagenic effects. Previous studies have shown that rosemary- and thyme-derived antioxidants can effectively retard lipid oxidation and delay rancidity in food systems, thereby improving the shelf life and product stability. However, the efficiency of these natural antioxidants is highly dependent on the food matrix, processing conditions, and application method, highlighting the importance of developing optimized strategies that allow their functional compounds to be retained during thermal treatment.^5–8^

Thermal processing methods such as roasting are known to induce complex chemical reactions that strongly influence both the nutritional and sensory attributes of legume-based foods. Roasting promotes the formation of aroma-active compounds through Maillard reactions, lipid oxidation, and other heat-induced pathways, while also affecting the stability and transformation of phenolic compounds.^9^ Although roasting can enhance flavor and overall acceptability, conventional roasting techniques often result in uneven heat distribution and prolonged exposure times, which may lead to the degradation of heat-sensitive bioactive compounds.^10^ Several studies have examined the effects of roasting temperature and time on the physicochemical properties of mung beans;^11–13^ however, traditional methods remain limited in terms of process control and uniform heating. Consequently, alternative roasting technologies that enable faster and more homogeneous heat transfer have attracted growing interest.^14^

In this context, the use of edible coatings combined with alternative roasting techniques represents a promising approach to modulating heat-induced chemical reactions and improving product quality. Coating materials rich in phenolic compounds may act as protective matrices during roasting, potentially influencing phenolic stability and volatile compound formation. Despite extensive research on rosemary and thyme as antioxidant sources, their application as edible coatings prior to roasting and their interaction with different roasting methods in legumes have been insufficiently explored. In particular, information about how coating-assisted roasting affects volatile compound profiles and underlying chemical transformations in mung beans is limited.

This study addresses this gap by investigating the combined effects of rosemary- and thyme-based edible coatings and different roasting methods on the physicochemical, bioactive, and volatile characteristics of mung beans. Microwave and air-fry roasting, which have recently emerged as alternatives to conventional oven roasting, were evaluated alongside traditional methods. To the best of our knowledge, this is the first study to examine the integrated use of phenolic-rich herb coatings applied before roasting, together with a comparative assessment of conventional and alternative roasting techniques. The aim of this work is to elucidate how coating-assisted roasting influences phenolic retention, antioxidant activity, and volatile compound formation, thereby providing chemical insight into the development of nutritionally and sensorially improved mung bean-based snack products.

## Materials and Methods

### Materials and Reagents

Mung beans, rosemary, and thyme were purchased from a local market in Izmir and stored at room temperature until use. Other reagents, including tetrabutylammonium hydroxide, acetic acid, methanol, acetonitrile, water, acetone, and sodium dihydrogen phosphate, were obtained from J. T. Baker (Philipsburg, NJ, USA). Distilled water used throughout the study was prepared by using a Milli-Q water purification system (Millipore, Milford, MA, USA).

Folin–Ciocalteu reagent, sodium carbonate (Na_2_CO_3_), Trolox standard, methanol, aluminum chloride, and sodium nitrate were supplied by Sigma-Aldrich (St. Louis, MO, USA). Gallic acid, Folin–Ciocalteu reagent, and 2,2-diphenyl-1-picrylhydrazyl (DPPH) were purchased from Sigma-Aldrich Pty Ltd. (Singapore City, Singapore). Analytical-grade methanol was obtained from Merck Ltd. (Ho Chi Minh City, Vietnam).

### Sample Preparation

#### Soaking and Boiling

A flowchart of the experimental procedure is presented in Figure 1. The soaking and boiling treatments were carried out according to a modified method described by Hefnawy.^15^ Briefly, the mung beans were washed thoroughly and soaked in water at 25 °C for 90 min. The soaked beans were then boiled in tap water at 100 °C by using a bean-to-water ratio of 1:10 (w/v) on a hot plate for 15 min. Doneness was determined based on the cooking time.

**Figure 1:**
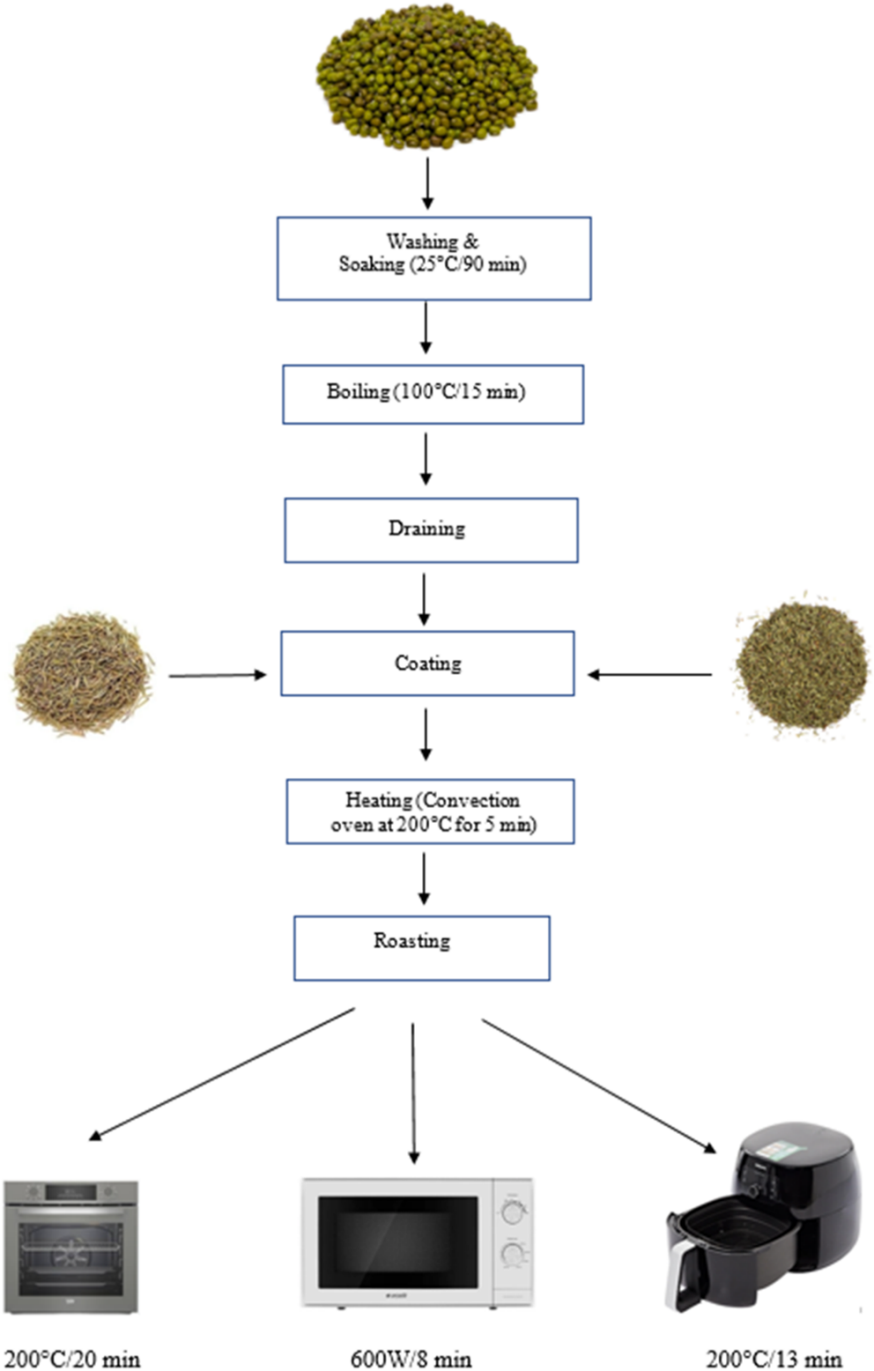
Flowchart of the roasting process of mung bean samples.

Boiling was applied as a pretreatment to ensure uniform hydration of the beans. Although boiling resulted in softening of the kernels, subsequent roasting led to surface dehydration, producing a texture characterized by a soft interior and a crispy outer layer. After boiling, the samples were drained and prepared for further processing.

The sample groups were Control-Oven (C-OV), Control-Microwave (C-MW), and Control-Airfryer (C-AF); Rosemary-Oven (R-OV), Rosemary-Microwave (R-MW), and Rosemary-Airfryer (R-AF); and Thyme-Oven (T-OV), and ThymeMicrowave (T-MW), and Thyme-Airfryer (T-AF).

#### Coating

Two separate coating materials, rosemary and thyme, were applied individually and not in combination. The coating procedure was adapted from the method described by Joray and Rayas-Duarte^16^ with minor modifications. Each experimental run was conducted using 300 g of mung beans, to which 30 g of either rosemary or thyme was added, corresponding to a coating level of 10% (w/w) based on the mung bean mass. Following boiling, the moist beans were uniformly sprinkled with the respective herb powder to ensure a complete surface coverage. The coated beans were spread on greaseproof paper and gently mixed to achieve a homogeneous coating. Subsequently, the coated mung beans were heated in a convection oven at 200 °C for 5 min to facilitate adhesion of the coating material.

#### Roasting

Mung beans were roasted using three different methods: oven roasting, microwave roasting, and air-fry roasting. The initial moisture content of the boiled mung beans was approximately 62% (wet basis, wb). For each roasting method, processing conditions were selected based on preliminary trials that evaluated changes in bean color and overall consumer acceptance. The final roasting parameters were optimized by testing various combinations of temperature, microwave power, and processing time while ensuring that the moisture content of the roasted beans was reduced to below 6% (wb).

Microwave roasting was performed by evenly spreading the beans on a rotating glass plate of a microwave oven (Arçelik MD 674, Türkiye) operated at 600 W for 8 min. For oven roasting, the beans were uniformly distributed on an aluminum tray and roasted in a conventional oven (Beko BFC 330 G, Türkiye) under natural convection conditions (without forced airflow) at 200 °C for 20 min. Air-fry roasting was carried out by placing the beans directly into the metal basket of an air fryer (Philips Airfryer XXL, HD9650/94, China) and roasting them at 200 °C for 13 min.

After being roasted, the mung beans were allowed to cool at room temperature for 30 min. The samples were then immediately ground into powder using a blender (Bosch MKM6000, Turkey). The resulting powders were transferred to sealed glass containers and stored at +4 °C. All analyses were completed within 24 h of roasting to minimize potential changes in volatile compounds. All time intervals were kept constant across all samples to ensure experimental consistency.

### Methods of Analysis

#### Moisture, Ash, and Protein Content

##### Proximate Composition

The moisture and ash contents of the samples were determined according to AOAC methods.^17^ Total nitrogen content of mung beans was measured using the micro-Kjeldahl method, and protein content was calculated by multiplying the nitrogen value by a conversion factor of 6.25.^18^ All protein and ash values (%) were expressed on a dry matter (DM) basis.

##### Preparation of Test Materials

Methanolic extracts were prepared by mixing 5 g of each sample with 80% (v/v) methanol and diluting to a final volume of 50 mL (1:10, w/v). The mixtures were stirred on a magnetic stirrer for 10 min and then homogenized at 8000 rpm for 5 min using an Ultra-Turrax homogenizer. The homogenates were centrifuged at 4000 rpm for 10 min (Hettich Universal 380R, Germany), and the resulting supernatants were collected and stored at 4 °C until further analysis.

##### Total Phenolic Content

The total phenolic content of mung bean samples was determined using the Folin–Ciocalteu method.^19^ Briefly, 0.5 mL of extract was mixed with 2.5 mL of the Folin-Ciocalteu reagent (0.2 N) and 2.0 mL of sodium carbonate solution (75 g/L). After incubation for 2 h in a dark and humidified environment, the absorbance was measured at 765 nm. Total phenolic content was calculated using a gallic acid calibration curve and expressed as milligrams of gallic acid equivalents (GAE) per 100 g of dry matter (DM).

##### Total Flavonoid Content

Total flavonoid content was determined using the aluminum chloride colorimetric assay.^20^ One milliliter of sample extract was mixed with 0.4 mL of sodium nitrate solution (50 g/L) and allowed to react for 6 min. Subsequently, 0.4 mL of an aluminum chloride solution (100 g/L) was added. After 6 min, 4 mL of a sodium hydroxide solution (40 g/L) was added, and the mixture was diluted to 10 mL with methanol. Following a 15 min incubation period, absorbance was measured at 510 nm by using a spectrophotometer (Shimadzu UV-1700, Kyoto, Japan). Quercetin was used as the reference standard, and results were expressed as milligrams of quercetin equivalents (QE) per 100 g of DM.

##### Total Antioxidant Activity

The antioxidant activity of mung bean extracts was evaluated using the DPPH radical scavenging assay according to Nguyen and Chuyen.^21^ Briefly, 2 mL of the extract (200 μL/mL) was mixed with 4 mL of 0.1 mM DPPH solution. The reaction mixture was incubated in the dark at room temperature for 30 min, after which the absorbance was measured at 527 nm using a spectrophotometer (V-630, Jasco, Japan). A blank containing methanol and a DPPH solution was prepared similarly. DPPH radical scavenging activity was calculated as a percentage based on the relationship between scavenging capacity and extract concentration.

##### Color Measurement

Color parameters of the snack samples were measured using a chromameter (Konica Minolta CR-400, Japan) after calibration with a standard white reference plate. Approximately 5 g of each sample was placed in a Petri dish, and measurements were taken at six different positions. Color values were recorded as L* (lightness, 0–100), a* (red to green), and b* (yellow–blue).

##### Determination of Volatile Compounds

Volatile compounds were analyzed by gas chromatography–mass spectrometry using a GC–MS system (Shimadzu GC-MS-QP2010, Japan) following the method of Coskun et al.^22^ Volatiles were extracted by solventless solid-phase microextraction (SPME) by using a fused silica CAR/PDMS fiber. Separation was performed on a Restek Rx-5Sil MS column (30 m × 0.25 mm inner diameter, 0.25 μm film thickness) with helium as the carrier gas at a flow rate of 1.61 mL/min.

Splitless injection was carried out at an injector temperature of 250 °C. The oven temperature program was as follows: initial temperature of 40 °C was held for 2 min, increased to 250 °C at a rate of 4 °C/min, and held for 5 min. Samples were equilibrated and exposed to the SPME fiber at 60 °C for 30 min, followed by desorption at 250 °C. Mass spectra were recorded at an ionization energy of 70 eV.

Volatile compounds were identified based on retention indices calculated using a C10–C26 *n*-alkane series and by comparison of mass spectra with Wiley, NIST, Tutor, and FFNSC libraries, with confirmation using 30 authentic standards. Semiquantification was performed using 1-octen-3-ol as an internal standard, and results were expressed as μg/100 g DM.^22^ Response factors were calculated based on the intensity ratio of each compound relative to the internal standard, and peak areas were adjusted accordingly. Compound concentrations were calculated using eq 1:

1 $$C_{I}=\frac{A_{I}}{A_{\mathrm{STD}}}\times C_{\mathrm{STD}}\times \mathrm{RF}\times \mathrm{CF}$$

*C*_*I*_: concentration of volatile compound; *A*_*I*_: peak area of volatile compound; *A*_STD_: peak area of internal standard; *C*_STD_: concentration of internal standard; *RF*: response factor; and *CF*: calculation factor.

### Statistical Analysis

All roasting experiments and analytical measurements were conducted in triplicate, and the results were expressed as mean ± standard deviation. Statistical analyses were performed using SPSS version 20.0 (SPSS Inc., Chicago, IL, USA). Differences among mean values were evaluated using one-way analysis of variance (ANOVA), followed by Duncan’s multiple range test at a significance level of *P* < 0.05.

Multivariate statistical analyses, including Principal Component Analysis (PCA) and Hierarchical Cluster Analysis (HCA), were performed using RStudio (R Project for Statistical Computing). Prior to multivariate analysis, the data were mean-centered and autoscaled to reduce the influence of variables with different magnitudes. PCA was applied to evaluate the relationships among samples and volatile compounds, and interpretation was based on the principal components explaining the majority of the total variance. HCA was carried out using Euclidean distance and Ward’s linkage method to determine similarity relationships and clustering patterns among samples.

## Results and Discussion

### Proximate Composition

The sample groups Control-Oven (C-OV), Control-Microwave (C-MW), and Control-Airfryer (C-AF); Rosemary-Oven (R-O), Rosemary-Microwave (R-MW), and Rosemary-Airfryer (R-AF); and Thyme-Oven (T-OV), ThymeMicrowave (T-MW), and Thyme-Airfryer (T-AF) were analyzed for moisture, protein, and ash content. Also, phenolic, flavanoid, and antioxidant activities, as inhibition % of DPPH were determined. The results were shown in Table 1.

**Table 1:** Results of Moisture, Protein, Ash Content, Phenolic, Flavanol, and Antioxidant Activities, Expressed as Inhibition % of DPPH, for the Mung Bean Samples^a^,^b^

sample	moisture content (%)	ash (%)	protein (%)	total phenolic content (mg GAE/100 g DM)	total flavonoid content (mg QE/100 g DM)	DPPH inhibition (%)
C-OV	4.80 ± 0.05^d^	4.0 ± 0.13^c^	23.28 ± 0.02^a^	777.0 ± 0.05^a^	11.50 ± 0.01^a^	16.70 ± 0.05^a^
C-MW	4.43 ± 0.02^c^	2.7 ± 0.15^a^	24.27 ± 0.05^a^	787.0 ± 0.05^a^	21.00 ± 0.02^b^	17.40 ± 0.03^b^
C-AF	2.72 ± 0.05^b^	3.0 ± 0.18^b^	25.25 ± 0.04^b^	1087.0 ± 0.08^b^	34.70 ± 0.06^c^	20.70 ± 0.02^c^
R-OV	5.31 ± 0.02^e^	3.3 ± 0.11^b^	23.28 ± 0.02^a^	1427.0 ± 0.00^c^	77.00 ± 0.04^d^	41.20 ± 0.05^d^
R-MW	5.25 ± 0.03^e^	3.7 ± 0.12^b^	24.28 ± 0.04^a^	1447.0 ± 0.02^d^	98.50 ± 0.05^e^	42.00 ± 0.04^d^
R-AF	2.50 ± 0.01^a^	3.3 ± 0.09^b^	25.96 ± 0.05^b^	1977.0 ± 0.01^e^	108.50 ± 0.08^f^	46.60 ± 0.05^e^
T-OV	5.56 ± 0.02^e^	3.5 ± 0.10^b^	23.25 ± 0.06^a^	897.6 ± 0.09^f^	35.74 ± 0.07^c^	26.86 ± 0.09^f^
T-MW	5.22 ± 0.04^e^	2.7 ± 0.12^a^	24.65 ± 0.04^a^	1447.0 ± 0.02^d^	40.70 ± 0.04^g^	27.80 ± 0.02^g^
T-AF	2.24 ± 0.04^a^	3.0 ± 0.11^b^	25.55 ± 0.05^b^	1975.6 ± 0.03^e^	55.00 ± 0.03^h^	28.40 ± 0.03^h^

a Superscript letters (a–h) mean that in the same column followed by different letters are significantly different (*p* < 0.05).

b Control-Oven (C-OV), Control-Microwave (C-MW), and Control-Airfryer (C-AF); Rosemary-Oven (R-OV), Rosemary-Microwave (R-MW), and Rosemary-Airfryer (R-AF); and Thyme-Oven (T-OV), ThymeMicrowave (T-MW), and Thyme-Airfryer (T-AF).

During roasting, the target moisture content was set below 6%. The uncoated oven-roasted sample (C-OV) exhibited a moisture content of 4.80%, whereas significantly lower values were observed in the C-MW and C-AF groups (*P* < 0.05). Among the roasting methods, air frying resulted in moisture contents that were significantly different from those obtained by microwave and oven roasting (*P* < 0.05). In general, coated samples exhibited higher moisture contents than uncoated samples, which can be attributed to the presence of rosemary and thyme, likely acting as partial barriers to moisture loss.

Previous studies have reported that the moisture content of mung beans is markedly affected by processing conditions. Moisture values of raw, infrared-processed, and conventionally cooked mung beans were reported as 10.43, 9.89, and 10.23%, respectively,^23,24^ while roasting at different time intervals resulted in much lower moisture contents (0.40–0.49%).^13^ Similar moisture-related variations have also been reported for coated oilseeds, where coating type significantly influenced water retention.^25^ In addition, increasing roasting temperature and time has been shown to reduce moisture content while relatively increasing crude protein, ash, and fat contents in mung bean flours.^26^ Cooking and dehulling processes were also reported to slightly modify ash and protein contents in mung beans.^27^

In the present study, the ash contents of mung bean samples ranged from 2.7 to 4.0%, which is in agreement with values reported in the literature. No statistically significant differences were observed among most samples, except for the T-MW, T-OV, and C-OV groups (*P* > 0.05). These findings indicate that neither the coating nor the roasting method caused pronounced changes in ash content under the applied conditions.

Comparable ash contents have been reported for microwave-roasted mung beans (2.10–2.91%) depending on roasting time and power,^13^ as well as for roasted mung beans compared to control samples.^28^ Similar trends were also observed in infrared-processed and conventionally cooked mung beans.^29^ Higher ash contents reported in some coated products were attributed to the mineral contribution of added coating materials.^29^ Furthermore, microwave roasting has been shown to result in lower mineral losses than boiling, suggesting improved mineral retention compared to conventional cooking methods.^30^ Overall, the ash contents obtained in this study fall within the reported range for roasted legumes (2.5–4.0%) and support the view that roasting, particularly under controlled conditions, does not adversely affect mineral composition.^31^Protein contents of the samples were found to be 23.28% (DM), 23.28% (DM), and 23.25% (DM) for the C-OV, R-OV, and T-OV groups, respectively. Air-fry roasting had a statistically significant effect on protein content (*P* < 0.05). Although rosemary- and thyme-coated samples exhibited slightly higher protein contents compared to the control, these differences were not statistically significant (*P* > 0.05).

Dahiya et al.^32^ emphasized the importance of processing studies on mung beans due to the limited literature available for this protein-rich legume. Mung bean (*V. radiata* L.) has attracted increasing attention owing to its high-quality protein content, reported to range between 14.6 and 32.6% among legumes.^33^ In line with the present findings, protein contents of 24.51, 24.89, and 24.36% were reported for infrared-processed, raw, and conventionally cooked mung beans, respectively.^29^ Du et al.^34^ reported a total protein content of 23.73% (dry basis) for mung beans, which was consistent with values reported in previous studies. They also highlighted that mung beans contain significantly higher amounts of high-quality protein compared with other legumes. Furthermore, Miftakhussolikhah et al.^28^ demonstrated that different processing methods, including blanching, steaming, and roasting, significantly influenced protein content, with roasting yielding the highest values. This effect was attributed to the absence of water during roasting, which limits the solubilization and loss of hydrophilic proteins. In contrast, steaming and boiling promote protein denaturation and leaching, resulting in lower measured protein contents. Similar trends have been reported for mung bean-based snacks and other legumes, where roasting preserved or slightly increased protein content compared to soaking or wet heat treatments.^1,35^

### Bioactive Properties

Phenolic and flavonoid contents as well as antioxidant activity expressed as DPPH inhibition (%), of the mung bean samples are presented in Table 1. Total phenolic content (TPC) differed significantly among the control and coated samples and was markedly affected by both coating application and roasting method (*P* < 0.05). Coated samples exhibited significantly higher phenolic contents than the control, with rosemary-coated samples showing the highest values among all groups. Microwave roasting also resulted in a significant increase in phenolic content (*P* < 0.05); however, the highest TPC values were obtained in air-fried samples across all formulations (control, rosemary-, and thyme-coated). This enhancement can be attributed both to the naturally high phenolic content of rosemary and to the favorable impact of air frying as a thermal processing method.

Previous studies have reported that processing techniques such as heating, germination, fermentation, and other thermal treatments can substantially alter polyphenol profiles in legumes.^36^ Similarly, air frying of Brassica vegetables at 160 °C for 10 min resulted in the highest total phenolic content and antioxidant activity, which was attributed to peroxidase inactivation at elevated temperatures, thereby reducing pro-oxidant activity.^37^ In a study evaluating roasting, microwaving, and steaming of black gram, the highest TPC value (424.35 mg/100 g dry basis) was observed after roasting.^38^ Zhang et al.^12^ reported that roasting mung beans at temperatures ranging from 110 to 200 °C affected phenolic content differently depending on the processing method, noting that cooking caused greater phenolic losses compared to steaming and roasting, although bound phenolics increased after cooking.

Total flavonoid content also differed significantly among roasting methods and coating treatments (*P* < 0.05). Flavonoid levels increased after microwave and air-fry roasting compared to oven roasting, with the highest flavonoid content (108.50 ± 3.08 mg QE/100 g DM) observed in rosemary-coated mung beans, particularly in the R-AF group. This increase is primarily attributed to the rich phenolic and antioxidant composition of rosemary. Previous studies have consistently reported higher antioxidant and phenolic contents in rosemary compared to thyme.^39^ In thyme-coated and control samples, air-fry roasting resulted in significantly higher flavonoid contents than other roasting methods (*P* < 0.05).

Antioxidant activity, expressed as DPPH radical scavenging capacity (% inhibition), is also presented in Table 1. The lowest antioxidant activity was observed in the C-OV group (16.70%), while the highest value was detected in the R-AF sample (46.60%). Both microwave and air-fry roasting led to significant increases in antioxidant activity compared to oven roasting (*P* < 0.05), with air frying showing the most pronounced positive effect. These findings indicate that air frying, as a domestic cooking method, effectively preserves or enhances antioxidant quality in mung bean products.

Consistent with these results, Kim et al.^100^ reported increases in phenolic and antioxidant contents of mung beans with increasing roasting temperature and time. They observed total polyphenol contents of 4.81–7.71 mg of GAE/g and total flavonoid contents of 2.46–3.05 mg of CE/g in roasted mung bean flours. Additionally, DPPH radical scavenging activity ranged between 174.41 and 346.70 mg TE/100 g, while ABTS radical scavenging activity varied from 274.39 to 430.02 mg TE/100 g.^26^ Alptekin and Bölek^13^ similarly reported increased antioxidant activity during roasting, which was attributed to the formation of new antioxidative compounds through Maillard reactions. Comparable trends were observed in black gram, where higher roasting temperatures, power levels, and processing times significantly increased antioxidant and phenolic contents due to the generation of Maillard reaction products.^38^ In the same study, microwave roasting yielded the highest antioxidant capacity compared to steaming, although differences were not statistically significant.

Song et al.^11^ demonstrated that roasting at 110 °C for 30 min resulted in the highest DPPH and ABTS radical scavenging activities, xanthine oxidase inhibition, and total phenolic and flavonoid contents. Alptekin and Bölek^13^ further reported that antioxidant capacity reached 39.42 ± 0.14% after microwave roasting at 600 W for 20 min, compared to 33.32 ± 0.22% at 600 W for 8 min, while significantly lower values (0.12 ± 0.09%) were observed after roasting at 130 °C for 10 min in a fluidized bed dryer. Similarly, the highest phenolic content (285.13 ± 0.21 mg of GAE/100 g) was obtained after microwave roasting at 600 W for 20 min, followed by 230.26 ± 0.24 mg of GAE/100 g at 600 W for 8 min, whereas fluidized bed roasting resulted in lower phenolic levels (210.03 ± 0.32 mg of GAE/100 g). Based on these findings, microwave roasting was suggested as a rapid and effective method for enhancing phenolic, flavonoid, and antioxidant properties of mung beans.^13^ Sruthi et al.^14^ further emphasized that roasting outcomes vary depending on oven type, and microwave processing has been reported as the least destructive method for γ-aminobutyric acid formation in mung beans.^40^

### Color Evaluation of the Roasted Mung Beans

Color parameters of the roasted mung bean samples are listed in Table 2. Lightness (L*) values differed significantly among roasting methods (*P* < 0.05), and the application of rosemary and thyme coatings also had a statistically significant effect on L* (*P* < 0.05). In the control samples, lightness decreased in the air-fried (C-AF) and microwave-roasted (C-MW) groups compared to the oven-roasted control (C-OV), reflecting the intensified thermal impact of these processes. In contrast, coated samples generally exhibited L* values higher than those of the uncoated control, indicating that rosemary and thyme coatings partially mitigated surface darkening during roasting. However, following air frying, lightness decreased significantly in all sample groups compared to oven-roasted samples, suggesting that air frying promoted more pronounced browning reactions.

**Table 2:** Color Values of Mung Bean Samples (L*, a*, b*)­^a^,^b^

Sample	L*	a*	b*
C-OV	22.97 ± 0.72^a^	8.68 ± 0.0.72^a^	16.48 ± 0.84^a^
C-MW	25.58 ± 0.28^b^	8.78 ± 010^a^	18.93 ± 0.34^b^
C-AF	20.82 ± 0.15^c^	9.73 ± 0.24^b^	15.50 ± 0.18^c^
R-OV	27.09 ± 0.44^d^	8.25 ± 0.0.19^a^	18.64 ± 0.35^b^
R-MW	38.12 ± 0.32^e^	8.41 ± 0.08^c^	20.34 ± 0.01^d^
R-AF	20.93 ± 0.09^c^	8.83 ± 0.08^a^	19.17 ± 0.19^e^
T-OV	35.74 ± 0.12^g^	6.67 ± 0.04^d^	22.06 ± 0.13^f^
T-MW	33.09 ± 0.69^h^	6.52 ± 0.09^d^	22.28 ± 0.20^f^
T-AF	22.08 ± 0.29^a^	8.61 ± 0.09^a^	14.07 ± 0.52^e^

a Superscript letters (a–h) mean that in the same column, followed by different letters, are significantly different (*p* < 0.05).

b Control-Oven (C-OV), Control-Microwave (C-MW), and Control-Airfryer (C-AF); Rosemary-Oven (R-O), Rosemary-Microwave (R-MW), and Rosemary-Airfryer (R-AF); and Thyme-Oven (T-OV), ThymeMicrowave (T-MW), and Thyme-Airfryer (T-AF).

Redness–greenness (a*) values increased with microwave and air-fry roasting as well as with rosemary and thyme coatings, indicating a shift toward redder and darker color tones as a result of elevated processing temperatures. Nevertheless, no significant differences were observed among the a* values of C-OV, C-MW, R-OV, R-AF, and T-AF samples (*P* > 0.05), suggesting that the combined effects of the coating and roasting method did not uniformly influence redness across all treatments.

Yellowness (b*) values also showed significant variation among the C-OV, C-MW, and C-AF groups. After air frying, the b* values decreased, indicating a darker and less yellow appearance. This outcome is consistent with intensified thermal reactions leading to pigment degradation and browning, and was therefore expected.

Similar trends have been reported in previous studies, where L* values decreased while a* and b* values increased with prolonged cooking time and higher temperatures. For instance, paste color was reported to change from bright to dark grayish when heated at 100–130 °C for 30–70 min, accompanied by increased a* and b* values and decreased L* values.^41^ Alptekin and Bölek^13^ observed a decrease in lightness and an increase in redness and yellowness in mung beans roasted at 180, 360, and 600 W for 8, 14, and 20 min, attributing these changes to melanoidin formation resulting from nonenzymatic browning reactions. Similarly, Kim et al.^100^ reported reduced L* values and increased a* and b* values in roasted mung bean flours, consistent with the present findings. These color changes have been associated with caramelization reactions and the formation of melanoidins under reduced water activity conditions, with the browning index showing a strong positive correlation with roasting temperature.^14^

### Volatile Compounds of the Roasted Mung Beans

The differences observed in the volatile profiles of roasted mung beans can be attributed to roasting-induced thermal reactions and the contribution of herb-based coatings. Thermal processing promotes Maillard reactions and lipid-related transformations, leading to the formation of aroma-active compounds that influence the overall flavor perception.

The volatiles found in mung beans were grouped into seven main chemical groups, including alkanes, alcohols, aldehydes and ketones, acids and esters, pyridines and pyrroles, terpenes, and aromatic compounds. Volatiles and their concentrations were determined by GC–MS and given in Table 3. Major compounds in mung beans are limonene, β-pinene, 2,5-dimethylpyrazine, methylpyrazine, o-xylene, 2-propanone, dodecane, and 4,6-dimethyldodecane.

**Table 3:** Volatile Compounds of Mung Bean (*V. radiata* L.) Samples (μg/100 g DM)^a^,^b^

**volatile compounds**	**RI**	**C-OV**	**C-MW**	**C-AF**	**R-OV**	**R-MW**	**R-AF**	**T-OV**	**T- MW**	**T-AF**
**alkanes**										
hexane	600	N.D.	2.92 ± 0.31^a^	42.78 ± 4.13^c^	67.81 ± 5.49^e^	108.21 ± 8.54^f^	30.65 ± 2.55^b^	30.15 ± 2.86^b^	55.89 ± 4.36^d^	60.64 ± 5.13^d,e^
2.4-dimethylheptane	890	N.D.	N.D.	N.D.	N.D.	N.D.	N.D.	N.D.	3.61 ± 0.13^a^	N.D.
4-methyldodecane	950	N.D.	N.D.	20.68 ± 1.12^b^	22.83 ± 1.06^b^	N.D.	N.D.	N.D.	13.41 ± 0.97^a^	N.D.
3-ethyl-3-methylheptane	960	6.87 ± 0.40^a^	5.25 ± 0.41^a^	53.05 ± 5.17^d^	26.44 ± 1.49^b^	N.D.	6.08 ± 0.49^a^	N.D.	36.87 ± 1.59^c^	N.D.
2-methylnonane	990	N.D.	N.D.	N.D.	4.54 ± 0.35^a^	N.D.	N.D.	N.D.	N.D.	N.D.
decane	1003	N.D.	N.D.	N.D.	N.D.	N.D.	N.D.	N.D.	19.31 ± 0.97^a^	N.D.
2.3.6.7-tetramethyloctane	1050	N.D.	N.D.	N.D.	N.D.	N.D.	8.97	N.D.	N.D.	N.D.
3.7-dimethyldecane	1100	N.D.	N.D.	13.44 ± 0.94^a^	23.61 ± 1.15^b^	N.D.	N.D.	N.D.	23.18 ± 1.26^b^	N.D.
5-isobutylnonane	1120	N.D.	N.D.	N.D.	N.D.	N.D.	N.D.	N.D.	7.12 ± 0.15^a^	N.D.
2-methylundecane	1180	N.D.	N.D.	N.D.	22.32	N.D.	N.D.	N.D.	N.D.	N.D.
6-ethylundecane	1190	5.13 ± 0.45^a^	5.04 ± 0.49^a^	33.36 ± 2.51^b,c^	52.38 ± 3.86^d^	N.D.	9.13 ± 0.85^a^	N.D.	25.62 ± 1.49^b^	N.D.
dodecane	1210	2.63 ± 0.40^a^	10.06 ± 0.95^b^	43.72 ± 2.85^d^	135.41 ± 8.61^e^	9.61 ± 0.85^b^	15.80 ± 1.10^c^	50.46 ± 3.26^d^	122.85 ± 7.51^e^	17.46 ± 0.96^c^
2.5-dimethylundecane	1220	3.55 ± 0.61^a^	2.50 ± 0.16^a^	24.28 ± 2.10^c^	45.26 ± 3.81^d^	N.D.	N.D.	N.D.	15.61 ± 0.77^b^	N.D.
4.6-dimethyldodecane	1270	21.14 ± 1.25^a^	36.31 ± 1.78^b,c^	118.38 ± 9.12^f^	78.23 ± 6.10^e^	27.83 ± 1.78^b^	31.03 ± 1.71^b^	38.12 ± 2.55^c^	70.55 ± 5.40^e^	59.88 ± 4.56^d^
tridecane	1305	N.D.	N.D.	N.D.	28.55 ± 1.15^b^	6.92 ± 0.46^a^	N.D.	N.D.	N.D.	N.D.
5-methyltetradecane	1440	1.79 ± 0.43^a^	8.47 ± 0.85^b^	59.93 ± 3.86^f^	47.36 ± 0.46^e^	7.45 ± 0.66^b^	N.D.	15.89 ± 0.97^c^	33.85 ± 2.49^d^	15.09 ± 0.80^c^
tetradecane	1455	15.63 ± 1.10^a^	27.20 ± 1.26^b,c^	14.34 ± 1.46^a^	46.65 ± 3.85^e^	23.10 ± 2.06^b^	32.74 ± 2.16^c,d^	75.17 ± 6.46^f^	78.82 ± 5.06^f^	16.48 ± 1.56^a^
hexadecane	1619	2.63 ± 0.16^a^	9.80 ± 0.85^d^	N.D.	N.D.	7.88 ± 0.74^c^	5.06 ± 0.49^b^	2.89 ± 0.06^a^	4.79 ± 0.41^b^	2.56 ± 0.03^a^
**total**		59.37	107.55	403.28	578.56	191.00	139.46	212.28	511.48	172.11
**alcohols**										
ethanol	830	N.D.	2.25 ± 0.13^a^	N.D.	15.53 ± 1.13^d^	10.31 ± 0.89^c^	14.15 ± 1.03^d^	6.17 ± 0.47^b^	N.D.	16.23 ± 0.81^d^
nopol	1200	N.D.	N.D.	N.D.	N.D.	5.89 ± 0.87^a^	N.D.	N.D.	N.D.	N.D.
1-octen-3-ol	1255	533.33 ± 8.55^a^	533.33 ± 8.55^a^	533.33 ± 8.55^a^	533.33 ± 8.55^a^	533.33 ± 8.55^a^	533.33 ± 8.55^a^	533.33 ± 8.55^a^	533.33 ± 8.55^a^	533.33 ± 8.55^a^
terpinen-4-ol	1280	N.D.	N.D.	N.D.	15.49 ± 0.43^c^	15.59 ± 0.46^c^	6.17 ± 0.53^a^	N.D.	N.D.	9.89 ± 0.87^b^
2-isopropyl-5-methyl-1-heptanol	1380	N.D.	N.D.	N.D.	N.D.	5.92 ± 0.56^a^	21.55 ± 1.15^b^	N.D.	N.D.	N.D.
endoborneol	1450	N.D.	N.D.	N.D.	29.07 ± 1.06^c^	22.71 ± 0.89^b^	8.97 ± 0.83^a^	N.D.	6.19 ± 0.53^a^	N.D.
**total**		533.33	535.58	533.33	593.42	593.75	584.17	539.50	539.52	559.45
**aldehydes& ketones**										
furfural	830	2.48 ± 0.44^a^	N.D.	6.37 ± 0.56^a^	N.D.	N.D.	45.29 ± 4.01^b^	N.D.	N.D.	82.50 ± 6.87^c^
hexanal	1080	1.16 ± 0.13^a^	2.08 ± 0.13^a,b^	5.17 ± 0.04^c^	7.58 ± 0.63^d^	6.32 ± 0.52^c,d^	7.67 ± 0.43^d^	N.D.	N.D.	9.51 ± 0.76^d,e^
6-methyl-5-hepten-2-one	1343	N.D.	N.D.	N.D.	N.D.	24.21 ± 1.16^a^	N.D.	N.D.	N.D.	N.D.
nonanal	1399	6.41 ± 0.16^a^	5.26 ± 0.20^a^	16.44 ± 1.08^d^	N.D.	9.11 ± 0.76^b^	18.42 ± 1.04^d^	5.78 ± 0.36^a^	12.40 ± 1.02^c^	27.76 ± 2.04^e^
2-propanone	1415	45.14 ± 2.46^a^	66.13 ± 5.13^b^	96.01 ± 8.79^c^	92.74 ± 8.45^c^	146.23 ± 10.39^d^	99.91 ± 8.16^c^	40.55 ± 3.93^a^	184.24 ± 10.46^e^	98.75 ± 8.01^c^
benzaldehyde	1543	N.D.	N.D.	9.89 ± 0.49^a^	26.16 ± 2.03^c^	N.D.	20.34 ± 1.60^b^	N.D.	N.D.	32.05 ± 2.03^d^
**total**		55.19	73.47	133.88	126.48	185.87	191.63	46.33	196.64	250.57
**acids and esters**										
ethyl acetate	610	N.D.	N.D.	N.D.	N.D.	7.78 ± 0.56^a^	N.D.	N.D.	N.D.	N.D.
ethanoate < hexyl->	1010	N.D.	N.D.	11.69 ± 0.87^a^	N.D.	N.D.	N.D.	N.D.	N.D.	N.D.
butyl hexanoate	1180	N.D.	N.D.	N.D.	N.D.	N.D.	13.52 ± 0.66^a^	N.D.	N.D.	N.D.
bornyl acetate	1285	N.D.	N.D.	N.D.	N.D.	13.37 ± 0.16^a^	N.D.	N.D.	N.D.	N.D.
4-terpinenyl acetate	1335	N.D.	N.D.	N.D.	N.D.	N.D.	N.D.	14.89 ± 0.91^b^	15.95 ± 0.93^b^	5.52 ± 0.09^a^
2-methoxydecanoic acid	2011	4.89 ± 0.21^a^	4.26 ± 0.36^a^	N.D.	N.D.	N.D.	N.D.	N.D.	14.07 ± 0.89^b^	N.D.
**total**		4.89	4.26	11.69	0	21.15	13.52	14.89	30.02	5.52
**pyridines& pyrroles**										
pyridine	670	N.D.	N.D.	N.D.	81.59 ± 4.56^a^	N.D.	N.D.	N.D.	N.D.	N.D.
pyrazine	720	N.D.	N.D.	N.D.	N.D.	25.76 ± 2.43^b^	4.92 ± 0.56^a^	N.D.	N.D.	137.42 ± 11.79^c^
methylpyrazine	850	13.89 ± 1.13^a^	24.27 ± 0.89^a,b^	139.45 ± 7.13^d,e^	110.63 ± 4.69^d^	30.26 ± 0.89^b,c^	180.99 ± 10.46^f^	N.D.	N.D.	178.46 ± 14.63^f^
ethylpyrazine	920	N.D.	N.D.	31.49 ± 1.95^b^	N.D.	11.59 ± 1.01^a^	29.82 ± 2.33^b^	N.D.	N.D.	35.35 ± 1.96^b^
2.5-dimethylpyrazine	940	11.23 ± 0.87^a^	23.44 ± 1.12^b^	184.09 ± 10.56^e^	123.91 ± 11.79^d^	41.22 ± 0.96^c^	174.99 ± 16.14^e^	N.D.	N.D.	186.59 ± 11.76^e^
4.6-dimethylpyrimidine	970	N.D.	N.D.	17.34 ± 1.20^b^	N.D.	N.D.	14.61 ± 1.09^a^	N.D.	N.D.	17.33 ± 1.26^b^
2-ethyl-6-methylpyrazine	1040	2.17 ± 0.16^a^	9.46 ± 0.56^b,c^	18.55 ± 0.61^e^	13.14 ± 0.87^d^	11.35 ± 0.55^c,d^	20.79 ± 0.78^e^	N.D.	N.D.	19.49 ± 1.03^e^
trimethylpyrazine	1041	N.D.	N.D.	71.60 ± 0.79^a^	70.79 ± 0.57^b^	N.D.	63.24 ± 0.65^a^	N.D.	N.D.	62.71 ± 0.87^a^
2.6-dimethyl-3-ethylpyrazine	1050	N.D.	N.D.	53.22 ± 5.12^d^	27.97 ± 1.54^b^	7.36 ± 0.13^a^	53.75 ± 4.29^d^	N.D.	N.D.	38.76 ± 1.59^c^
methylpyrrole	1482	N.D.	N.D.	N.D.	N.D.	N.D.	4.84 ± 0.23^a^	N.D.	N.D.	16.67 ± 0.89^b^
**total**		27.29	57.17	515.74	428.03	120.18	547.95	0	0	692.78
**terpenes**										
DELTA.3-carene	1010	N.D.	N.D.	N.D.	N.D.	7.28 ± 0.55^a^	N.D.	N.D.	N.D.	N.D.
α- pinene	1021	1.11 ± 0.56^a^	3.87 ± 0.16^b^	4.93 ± 0.26^b^	37.46 ± 3.01^d^	49.54 ± 4.56^e^	N.D.	N.D.	N.D.	11.14 ± 1.04^c^
camphene	1066	N.D.	N.D.	N.D.	8.09 ± 0.40^b^	5.28 ± 0.29^a^	N.D.	N.D.	N.D.	N.D.
sabinene	1100	N.D.	N.D.	N.D.	2.02 ± 0.26^a^	N.D.	N.D.	N.D.	N.D.	N.D.
β-pinene	1102	1.89 ± 0.56^a^	5.62 ± 0.63^b,c^	15.53 ± 1.03^e^	24.73 ± 2.07^f^	11.29 ± 0.56^d,e^	8.12 ± 0.46^c,d^	20.56 ± 2.00^f^	N.D.	32.12 ± 3.08^g^
β- myrcene	1149	N.D.	7.68 ± 0.46^b,c^	N.D.	50.27 ± 4.89^e^	49.72 ± 3.25^e^	9.97 ± 0.46^c^	3.79 ± 0.02^a^	10.55 ± 1.00^c^	34.32 ± 2.24^d^
limonene	1189	45.19 ± 2.56^b^	64.18 ± 5.14^c^	112.83 ± 8.50^d^	368.35 ± 12.56^f^	188.95 ± 8.69^e^	104.86 ± 5.26^d^	15.63 ± 1.03^a^	48.74 ± 2.69^b^	365.03 ± 15.63^f^
verbenone	1205	N.D.	11.16 ± 0.26^a^	N.D.	276.45 ± 9.16^a^	241.54 ± 8.96^c^	56.01 ± 5.13^b^	N.D.	N.D.	N.D.
γ- terpinene	1246	N.D.	N.D.	N.D.	N.D.	N.D.	N.D.	N.D.	N.D.	9.42 ± 0.89^a^
α- copaene	1504	N.D.	N.D.	N.D.	N.D.	8.63 ± 0.57^a^	N.D.	N.D.	N.D.	N.D.
camphor	1544	N.D.	N.D.	N.D.	54.90 ± 2.17^c^	33.01 ± 1.24^b^	8.92 ± 0.91^a^	N.D.	N.D.	6.99 ± 0.53^a^
linalool	1549	N.D.	N.D.	N.D.	69.06 ± 2.12^d^	52.37 ± 4.46^c^	38.96 ± 0.94^b^	18.64 ± 0.91^a^	100.78 ± 10.07^e^	37.14 ± 1.36^b^
α- humulene	1694	N.D.	N.D.	N.D.	N.D.	9.01 ± 0.98^a^	N.D.	N.D.	N.D.	N.D.
α- terpineol	1712	N.D.	N.D.	N.D.	65.61 ± 4.79^c^	59.30 ± 2.19^b,c^	17.07 ± 1.05^a^	N.D.	N.D.	N.D.
geraniol	1853	N.D.	N.D.	N.D.	N.D.	28.40 ± 1.76^a^	N.D.	N.D.	N.D.	N.D.
carvacrol	2196	N.D.	N.D.	N.D.	N.D.	N.D.	N.D.	260.45	532.99	250.73
**total**		48.19	81.35	133.29	956.94	744.32	243.91	319.07	693.06	746.89
**aromatic compounds**										
p-xylene	1128	N.D.	N.D.	N.D.	18.96 ± 10.78^a^	N.D.	N.D.	N.D.	N.D.	N.D.
o-xylene	1135	8.16 ± 0.56^a,b^	4.28 ± 0.47^a^	14.15 ± 1.13^c^	68.25 ± 4.79^g^	20.33 ± 1.16^d^	83.62 ± 4.69^h^	28.16 ± 1.16^e^	N.D.	52.13 ± 5.01^f^
1.3-ditert-butylbenzene	1249	N.D.	8.60 ± 0.80^a^	N.D.	N.D.	N.D.	19.04 ± 1.13^b^	N.D.	89.82 ± 6.17^c^	N.D.
dimethylstyrene < α-para->	1258	N.D.	N.D.	N.D.	58.74 ± 3.91^b^	10.49 ± 0.56^a^	N.D.	N.D.	N.D.	N.D.
**total**		8.16	12.88	14.15	145.95	30.82	102.66	28.16	89.82	52.13

a Superscript letters (a–i) indicate statistical significance between mung bean samples (*P* < 0.05).

b Note: Data represent the mean values and standard deviations from duplicate injections. RI Retention indices, ND not detected. Control-Oven (C-OV), Control-Microwave (C-MW), and Control-Airfryer (C-AF), Rosemary-Oven (R-O), Rosemary-Microwave (R-MW), and Rosemary-Airfryer (R-AF); and Thyme-Oven (T-OV), Thyme-Microwave (T-MW), and Thyme-Airfryer (T-AF).

Alkane compounds were detected in all samples; however, their concentrations varied significantly, depending on the applied processing method and the inclusion of rosemary and thyme. The alkanes do not contribute to odor as significantly as alcohols, aldehydes, acids, and ketones; however, they constitute an important group of compounds and provide evidence that the metabolism of flavor precursors was reported by researchers.^42,43^ The highest total alkane content was detected in R-OV (578.56 μg/100 g of DM), followed by T-MW (511.48 μg/100 g of DM) and C-AF (403.28 μg/100 g of DM), whereas C-OV (59.37 μg/100 g of DM) and R-MW (191.00 μg/100 g of DM) contained the lowest concentrations. It was observed that the oven-roasting method was the most effective for preserving alkane compounds in rosemary, whereas the microwave method was the most effective for thyme. Dodecane (135.41 μg/100 g DM in R-OV; 122.85 μg/100 g DM in T-MW) and 4,6-dimethyldodecane (118.38 μg/100 g DM in C-AF; 78.23 μg/100 g DM in R-OV) were the predominant compounds among all samples. In addition, tetradecane reached considerable levels in T-OV (75.17 μg/100 g of DM) and T-MW (78.82 μg/100 g of DM). On the other hand, 3-ethyl-3-methylheptane was found in higher amounts in C-AF (53.05 μg/100 g DM). This observation suggests that the applied processing method can lead to different concentrations of alkane compounds, depending on the presence or absence of rosemary and thyme.

Alcohols were detected in all samples. 1-Octen-3-ol (533.33 μg/100 g DM) was used as an internal standard in all samples. The concentration of 1-Octen-3-ol in mung beans was previously reported as 1211.7 peak area,^44^ 7.81 μg/100 g DM,^45^ and between 0.61–1.40 μg/100 g,^46^ respectively. The ethanol content of the rosemary-containing samples was found to be higher in oven (15.53 μg/100 g of DM) and microwave (10.31 μg/100 g of DM) roasted processes. It was observed that the total alcohol levels were elevated across all roasting treatments of the samples with rosemary.

The volatile aldehyde and ketone profile of mung bean samples varied depending on the processing method and the coating of rosemary and thyme. The total volatile concentration ranged from 55.19 μg/100 g of DM (C-OV) to 250.57 μg/100 g of DM (T-AF). 2-Propanone was determined as the predominant ketone compound, with concentrations ranging between 40.55 and 184.24 μg/100 g of DM. In all roasted processes of the control as well as thyme- and rosemary-coated samples, significantly higher levels of aldehyde and ketone compounds were detected, particularly under AF treatment. Hexanal and nonanal, which are well-recognized markers of lipid oxidation,^47,48^ were consistently detected in most of the samples. The highest concentrations were observed in T-AF, indicating that air frying treatment promoted more pronounced oxidative alterations compared to those in other processing methods. Roasting processing of mung bean promoted the formation of Maillard reaction products such as furfural and hydroxymethylfurfural (HMF). These compounds, generated during oven, microwave, and air frying treatments, likely contributed to the enhanced aroma and color, with the highest levels observed in air-fried samples, indicating that intense heat accelerates Maillard reactions.^49–51^ The detection of compounds such as methyl benzaldehydes and isophorone indicates the occurrence of carotenoid oxidation reactions.^9^ These reactions not only produce characteristic volatiles, including benzaldehyde derivatives, but also contribute to the visual changes observed during the roasting process. Consequently, they influence the aroma profile of mung bean products by enhancing roasted and nutty notes while simultaneously increasing oxidative volatiles. Overall, the concurrent oxidation of carotenoids during roasting plays a dual role, shaping both the aroma development and the visual appearance of the final product. Notably, this formation was more pronounced in the control and thyme-coated samples under AF treatment.

The formation of acids and esters in mung beans was dependent on the applied processing technique and the coating of rosemary and thyme. In control samples, the concentration of 2-methoxydecanoic acid was relatively low in C-OV (4.89 μg/100 g of DM) and C-MW (4.26 μg/100 g of DM), whereas the level of ethanoate (hexyl) in C-AF (11.69 μg/100 g of DM) was markedly higher. In our study, ethyl acetate was detected only in R-MW at 77.8 μg/100 g DM, whereas Xue et al.^46^ reported that the ethyl acetate content in all mung bean flours following *Lactobacillus* fermentation ranged from 0.55 to 1.35 μg/kg. This difference may be attributed to the different processing techniques applied to the mung beans, which can significantly influence the formation and accumulation of ethyl acetate. Among roasted samples, the absence of detectable compounds in R-OV contrasted with the considerably higher concentration observed in T-MW (30.02 μg/100 g DM), primarily attributed to 4-terpinenyl acetate and 2-methoxydecanoic acid.

The processing method and the rosemary and thyme coatings had a significant impact on the formation of pyridines and pyrroles. Control samples (except for C-AF) showed low totals (27.29–57.17 μg/100 g DM), whereas all AF-treated samples (C-AF, R-AF, T-AF) exhibited markedly higher pyridines and pyrroles concentrations (515.74–692.78 μg/100 g DM). The contents of 2,5-dimethylpyrazine and methylpyrazine were the highest volatile compound in samples (except for T-OV and T-MW). Similarly, in previous studies,^10,20^ 2,5-dimethylpyrazine was also among the most abundant volatile compounds detected. Methylpyrazine, 2.5-dimethylpyrazine, and pyrazine were the main contributors, indicating their formation through Maillard reactions. Strong sensory qualities are possessed by pyrazine, which is identified as a main Maillard reaction product that is produced by the Strecker degradation of leucine, isoleucine, and glycine.^52^ 2.5-dimethylpyrazine, in particular, imparts an individual baked aroma comparable to that of roasted peanuts or potatoes and plays a key part in both microwave and baking treatments. Additionally, pyrazine content rises during fermentation, impacting burnt, nutty/cocoa notes and acting as a primary fragrance ingredient in salted egg yolk, according to a recent study.^53^ Overall, the findings suggest that air frying (AF) significantly enhances the formation of Maillard-derived heterocyclic compounds, including pyridines, pyrroles, and pyrazines, shaping the characteristic aroma profile of mung beans. This enhanced formation in air-fried samples, compared to oven-roasted ones at the same nominal temperature (200 °C), is likely due to faster surface heating and more efficient moisture removal, which accelerate Maillard reactions at the bean surface.

The release of terpene compounds varies depending on the processing method and on the coating of rosemary and thyme. The concentrations of terpene compounds were notably higher in the rosemary (243.91–956.94 μg/100 g DM) and thyme (58.62–496.16 μg/100 g DM)-coated samples compared to the controls (48.19–133.29 μg/100 g DM). This outcome can be attributed to the richness of rosemary and thyme in terpene compounds, which substantially contribute to the enhancement of the volatile profile in coated samples. This observation is supported by previous research investigating the volatile profiles of rosemary^54–56^ and thyme,^57,58^ which consistently reported high levels of terpene compounds, thereby corroborating the present findings. Limonene (15.63–368.35 μg/100 g DM), linalool (18.64–100.78 μg/100 g DM), verbenone (11.16–276.45 μg/100 g DM), camphor (6.99–54.90 μg/100 g DM), and β-pinene (1.89–32.12 μg/100 g DM) were the prevalent compounds. The presence of carvacrol exclusively in thyme-coated samples is consistent with literature reports^59–62^ indicating the high carvacrol content of thyme and highlighting its significant impact on the aroma profile. In terms of roasting methods, OV was found to be the most effective in preserving terpene compounds in rosemary, whereas AF proved to be the most effective for thyme. Our results demonstrated that thermal processing significantly affected the terpene composition of mung beans, in line with previous findings on legumes. Studies on kidney beans and cowpeas reported losses of linalool, estragole, eucalyptol, and other terpenes under boiling, attributed to volatilization and thermal degradation.^51^ Conversely, γ-terpinene was shown to increase in chickpeas as a degradation product of limonene, contributing citrus-like notes. These observations corroborate the present findings, highlighting that terpenes in mung beans are highly sensitive to processing, with both degradation and transformation processes shaping their volatile profile.

Aromatic volatiles in mung beans varied notably with the processing method and with the coating of rosemary and thyme. Among them, o-xylene was abundant in roasted samples, particularly R-AF (83.62 μg/100 g DM) and R-OV (68.25 μg/100 g DM), and p-xylene appeared only in R-OV, suggesting dependency on specific roasting conditions. 1,3-di-*tert*-butylbenzene reached its maximum in T-MW (89.82 μg/100 g), reflecting microwave-induced transformations. Dimethylstyrene was detected mainly in R-OV (58.74 μg/100 g of DM) and R-MW (10.49 μg/100 g of DM). In the study on *Rosmarinus officinalis* L. essential oils from Spain, GC-MS analysis revealed that dimethylstyrene was present at relatively high levels, indicating that rosemary essential oil is a rich source of this styrene derivative.^63–65^ Overall, it can be concluded that the rosemary-coated samples exhibited higher levels of aromatic compounds.

Principal Component Analysis (PCA) was performed to evaluate the variation in volatile compounds among the different mung bean sample groups. As shown in the biplot (Figure 2), the first two principal components (PC1 and PC2) accounted for 35.6 and 25.1% of the total variance. Samples coated with rosemary (R-OV, R-MW, R-AF) clustered closely along the positive side of PC2, suggesting higher contents of specific volatiles such as limonene, α-pinene, 2-propanone, ethanol, and 2.5-dimethylpyrazine. In contrast, thyme-coated samples (T-OV, T-MW, and T-AF) and control samples (C-OV, C-MW, and C-AF) were more dispersed along PC1 and PC2, indicating differences in their volatile profiles.

**Figure 2:**
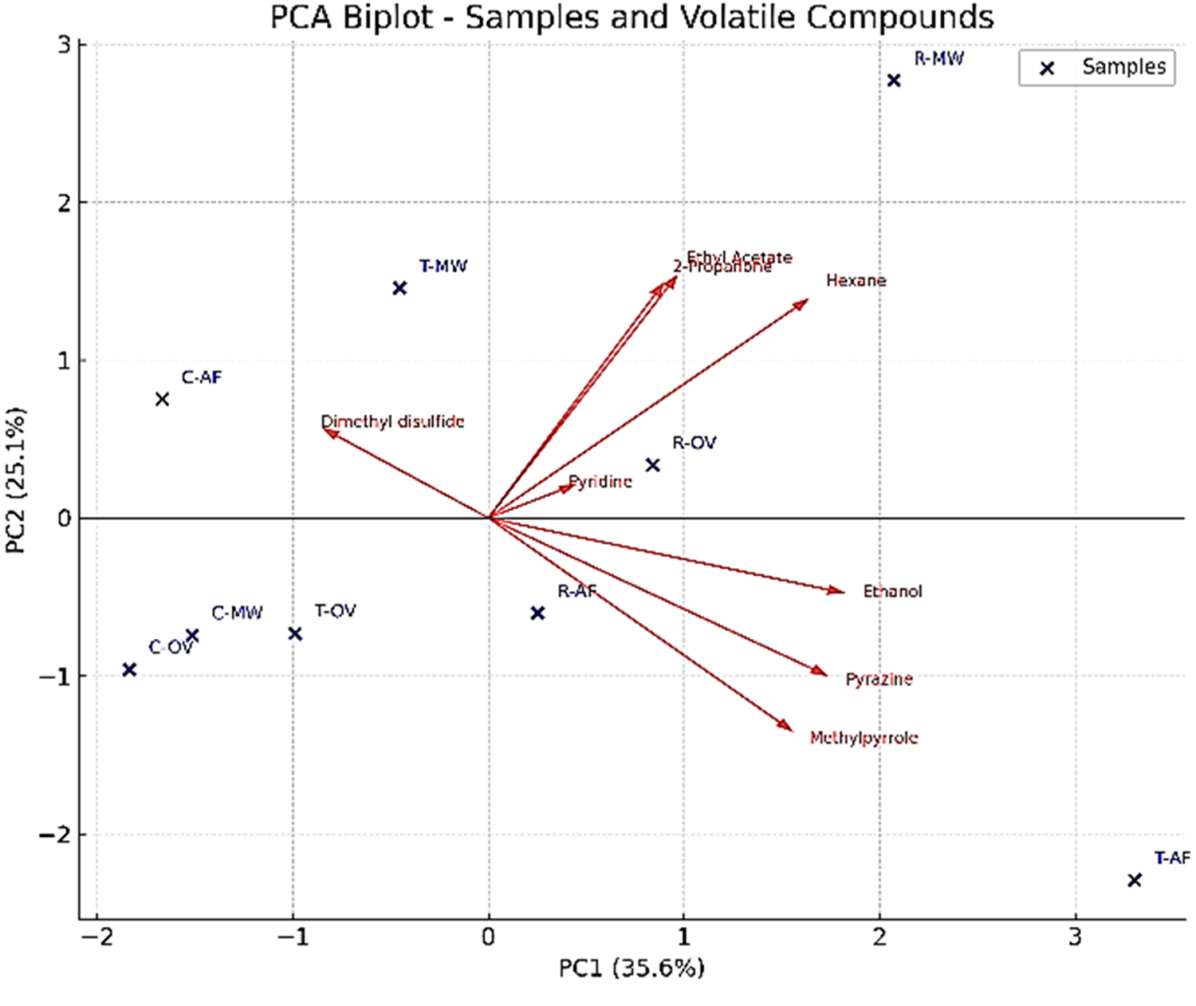
Principal component analysis (PCA) of mung bean samples.

The T-OV and T-MW samples, as well as the other sample groups, exhibited similar volatile profiles and, therefore, were clustered together in the hierarchical cluster analysis (HCA) dendrogram (Figure 3).

**Figure 3:**
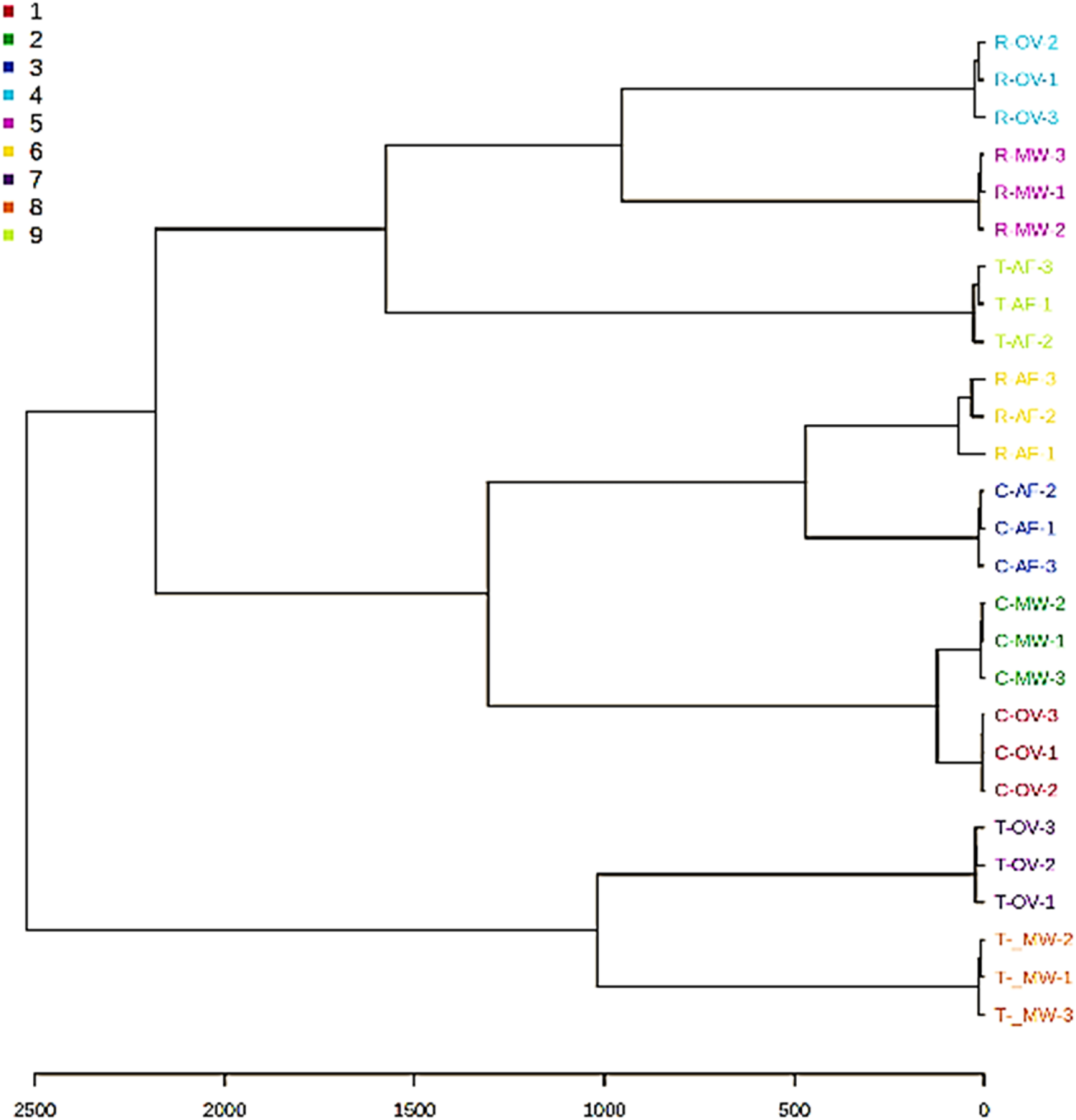
Hierarchical cluster analysis (HCA) of mung bean samples.

The mung bean samples were separated into two main clusters in PCA, reflecting differences in volatile compound profiles (Figure 4). The first cluster includes R-OV and R-MW, which showed similar profiles of volatile compounds such as caryophyllene, α-pinene, verbenone, α-terpineol, endoborneol, camphor, camphene, eucalyptol (1,8-cineol), p-cimene, terpinen-4-ol, and β-myrcene, both qualitatively and quantitatively. The second cluster comprises C-AF, T-AF, R-AF, T-MW, T-OV, C-OV, and C-MW, indicating that processing method and coating type substantially influence volatile composition. Among the major compounds, limonene, β-pinene, 2,5-dimethylpyrazine, methylpyrazine, o-xylene, and 2-propanone largely drive the separation of samples. Notably, R-OV exhibited the highest levels of sabinene, 2-methylnonane, 2-methylundecane, pyridine, p-xylene, and tridecane, which are associated with characteristic herbal and roasted notes. Overall, the PCA demonstrates that rosemary and thyme coatings, along with the choice of processing method, significantly shape the aroma profile of mung beans by altering key terpenes, pyrazines, and other volatile compounds.

**Figure 4:**
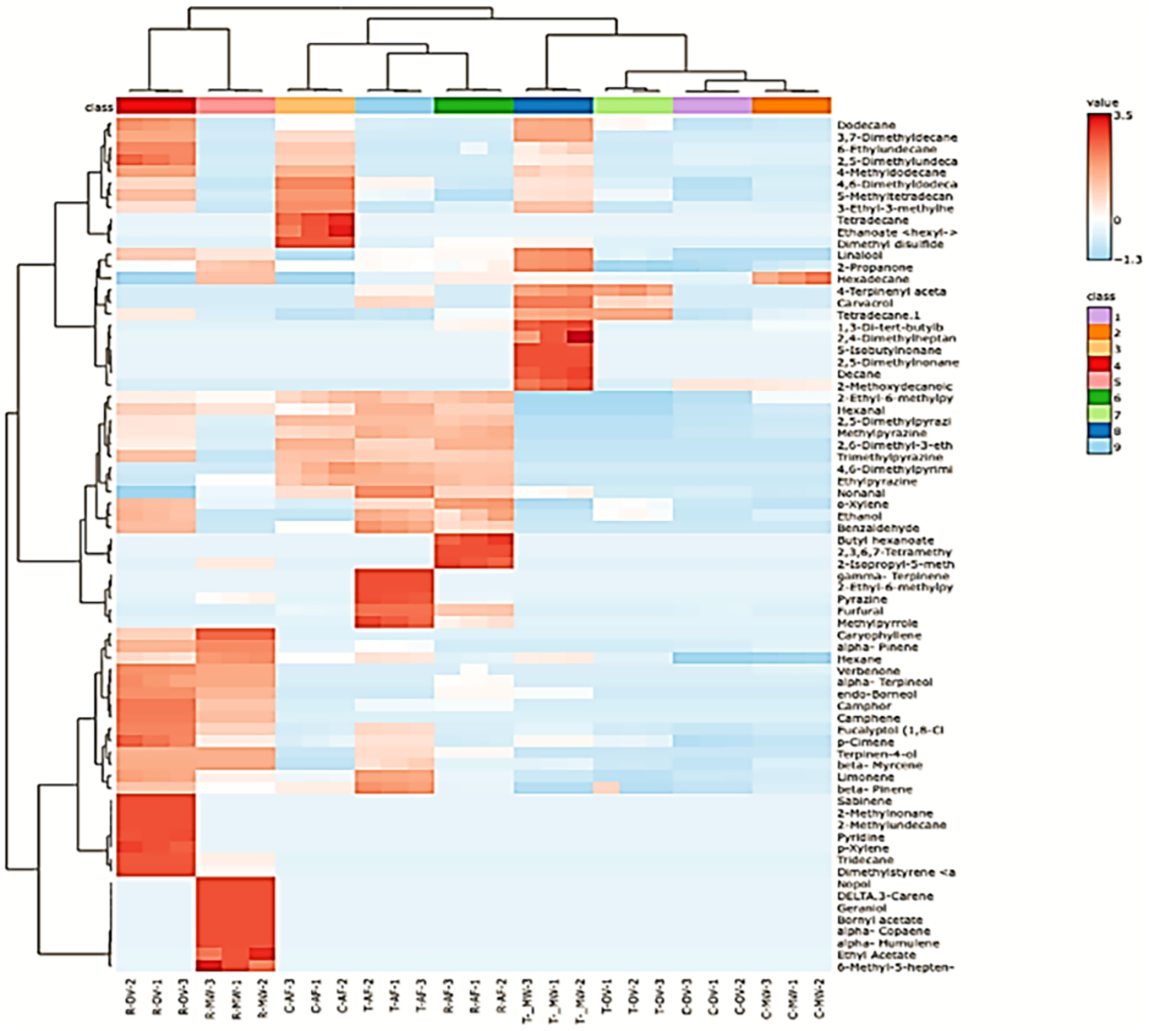
Heat map of volatiles from mung bean samples.

Specific volatile compounds serve as key discriminators among mung bean samples, as illustrated in Figure 5. Moreover, the type of coating and roasting processes appears to enhance the formation of certain volatiles while suppressing others. Among these, limonene, hexane, and nonanal were identified as dominant contributors to the overall volatile profile, reflecting pathways related to terpene biosynthesis and lipid oxidation.

**Figure 5:**
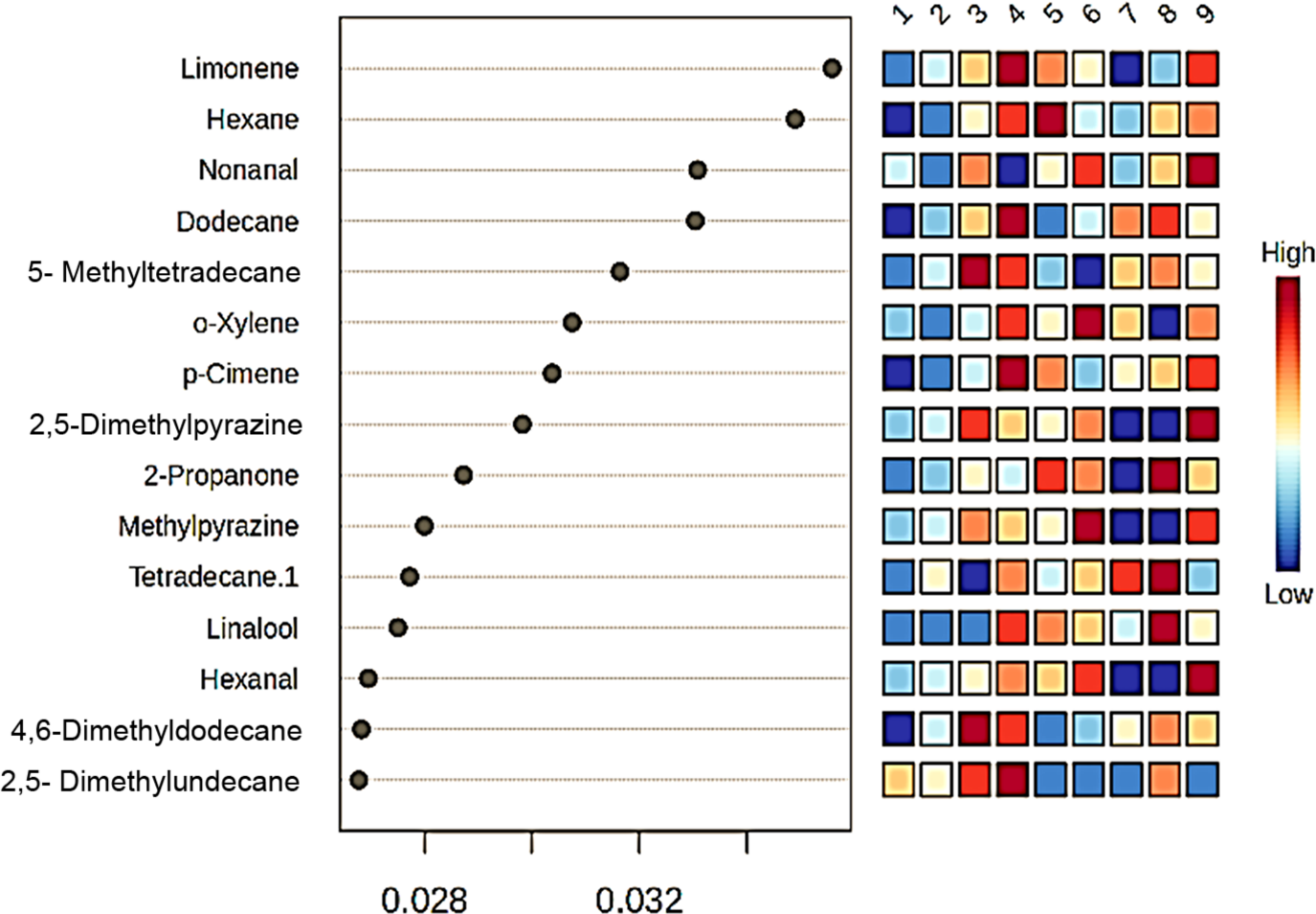
Variable importance in projection (VIP) score plot of volatile compounds in mung bean samples.

## Conclusion

This study shows that the combined use of edible herb coatings and different roasting techniques can improve the nutritional and functional quality of mung beans as a snack product. Among the tested methods, air-fry roasting resulted in higher phenolic, flavonoid, antioxidant, and protein contents than oven and microwave roasting, particularly when combined with a rosemary coating. This indicates that air frying is more effective in preserving or enhancing bioactive compounds during thermal processing. Although roasting caused a general decrease in lightness, the a* and b* color values were largely maintained across treatments.

Volatile compound analysis further demonstrated that both the roasting technique and coating type played a decisive role in aroma development. Rosemary-coated and air-fried samples showed higher levels of characteristic aroma-active compounds, such as limonene, nonanal, and hexane, suggesting that coating-assisted roasting can modulate flavor formation through heat-induced chemical reactions rather than simple physical changes.

A limitation of the present study is the lack of sensory evaluation. Since consumer perception is critical for snack products, future research should include sensory analysis to better relate instrumental volatile data to flavor perception and overall consumer acceptance.

## Data Availability

Deposit raw data tables for proximate/bioactive/GC–MS outputs and the PCA input matrix in SI or a repository.
